# Regulation of Prion Protein Expression: A Potential Site for Therapeutic Intervention in the Transmissible Spongiform Encephalopathies

**Published:** 2006-12

**Authors:** C. L. Haigh, D. R. Brown

**Affiliations:** *Department of Biology and Biochemistry, University of Bath, Bath, UK*

**Keywords:** prion, prnp, promoter, TSE

## Abstract

The Transmissible Spongiform Encephalopathies (TSEs) are a group of rare neurodegenerative diseases, which can be transmitted between members of the same species and possibly across different species. The link between the emergence of Bovine Spongiform Encephalopathy (BSE) and the new variant form of Creutzfedlt Jakob Disease (vCJD) has been the cause of much public concern. vCJD is the most widely known of the human TSEs but by no means the most common; inherited and sporadic forms are much more prevalent. The agent responsible for these diseases is a conformationally altered form of a normal cell surface glycoprotein, called the prion protein (PrP). The normal isoform must be present for the disease to progress, and disease incubation time decreases with increased PrP expression. There is still no cure for any of these diseases but recent advances in the understanding of how prion protein expression is regulated at the genetic level, and of exogenous factors modulating expression levels, may provide new insights into potential therapeutic targets for disease management by down regulation of cellular PrP levels.

## INTRODUCTION

The emergence of the TSEs is not a new event; however before the BSE epidemic in the 1980s their existence was virtually unknown. Human TSEs include, in addition to the widely known vCJD, hereditary forms of CJD, Gerstmann-Sträussler-Scheinker syndrome, fatal familial insomnia and Kuru. In addition, CJD can occur sporadically, possibly caused by somatic mutation in the prion gene. Sporadic and hereditary TSEs account for the majority of human cases (1). These diseases are characterised by the spongiform changes in the brain from which the diseases derived their name, and may be accompanied by plaques of insoluble amyloid-like protein (2).

TSE diagnosis is problematic. By the time of symptomatic presentation the disease is advanced, and there is currently no way to reverse the damage. Therefore early diagnosis is essential to maintain the quality of life of the patient. However, currently this is not possible since definitive diagnostic techniques require brain tissue post mortem. This difficulty diagnosing TSEs, accompanied by the long incubation periods reported for these diseases, has impacted on the health service in other areas including the risk of disease transmission through the use of human pituitary derived hormones, by cadaveric grafts, or by transmission from blood or blood products (3-8).

The NHS is further affected by the lack of knowledge on how vCJD can be transmitted. Studies have shown that the disease protein can become attached to surgical instruments so firmly that normal decontamination procedures are rendered useless. If these instruments are then used on further patients, the disease could be passed on through contact (6). This has been of considerable concern with tonsillectomies, where the tissue removed contains high numbers of leucocytes and shows positive immunohistochemistry for PrP^sc^ during disease (9). Further disciplines such as endoscopy (10), dentistry (11), and opthalmics (12) are also affected by the need to take extra measures to reduce the risk of CJD transmission between patients.

In addition to the problem of diagnosis there is currently no cure for prion diseases. Treatment focuses on managing the symptoms and making patients comfortable. A great deal of research has focused, ineffectually, on finding a cure. Pentosan sulphate/polysulphate is known to cure PrP^sc^ infected cells in vitro and so has been suggested as a potential treatment. Recently pentosan polysulphate was use to treat an eighteen year old (at the start of treatment) male with advanced vCJD (13). Drug delivery had to be by daily cerebrovascular infusion and some evidence of improvement in awareness and sleep cycles was detected in the patient. However, despite improvement, brain atrophy continued to progress, and, given the unpleasant administration process of the drug, investigations still continue into finding a better agent for treatment of these diseases.

### The Prion Protein

The agent responsible for the TSEs was identified as an infectious protein, and the term prion protein (PrP) devised to identify this proteinacious infectious particle (14). The infectious protein is now known as the scrapie isoform (PrP^sc^). PrP^sc^ is the disease related form of a normal cellular protein (PrP^c^). PrP^c^ is a glycophosphatidylinositol (GPI) anchored cell surface glycoprotein. It binds copper via a highly conserved repeat domain in its mainly unstructured N-terminal region, and its C-terminal structured region has high alpha helical content (15-17). When copper is bound to PrP^c^ it has been shown to have antioxidant function (18, 19), and so may function to protect cells against oxidative stress insults. However, other roles for PrP^c^ have been proposed including copper transport or storage (20, 21), protection against metal ion toxicity (22, 23), signal transduction (24-27), cellular adhesion (28, 29), and in promoting sleep continuity (30, 31). The PrP^sc^ isoform differs from the PrP^c^ isoform in that the structure has been altered to contain a higher beta-sheet conformation. In addition, PrP^sc^ cannot bind copper and lacks the associated anti-oxidant function.

The mechanism by which PrP^c^ is converted to PrP^sc^ is still a matter of debate. Conversion is thought to occur as a post-translational modification of PrP^c^, as opposed to a folding mistake during PrP^c^ synthesis. PrP^sc^ would act as a catalyst for this modification. What is certain is that endogenous PrP^c^ is required for conversion and so disease establishment. PrP^c^ knockout mice are resistant to infection with scrapie (32), and thus transmission of prion diseases and the resulting neurotoxicity requires the presence of PrP^c^ (33, 34). The level of PrP^c^ expression has been shown to influence the incubation period of the disease in mice (35, 36), and polymorphisms within the gene promoter alter PrP^c^ expression levels (37, 38). Hence, the role of the promoter and expression control elements may be fundamental in both disease pathogenesis and control.

### The Control of Prion Protein Expression

Regulation of protein expression is an essential function of all cells. A careful balance must be maintained for normal cellular homeostasis, and the ability to appropriately up or down-regulate specific proteins in response to stimuli is crucial to avoid damage to the individual cell or organ. Such is the importance of maintaining correct cellular expression levels that regulation occurs by mechanisms throughout the cell, acting on the gene itself, and all pathways through creation to destruction of the protein product. These regulatory mechanisms include; control of the transcription of mRNA from DNA by elements within the promoter sequence or up-stream of it; control of the translation of the mRNA by mRNA turnover (in turn influenced by the ability of the mRNA to form secondary structures to increase stability, by poly-adenylation, and by alternative splicing) or by micro RNAs (miRNAs) binding to sites within the mRNA sequence; and regulation of the protein itself by turnover mechanisms within the cell. The control of transcription is thought to be the most important factor for regulation of protein levels.

Eukaryotic promoter control is extremely complex. A pre-initiation complex (PIC) must assemble around the appropriate sequence of DNA, functioning to first uncoil supercoiled DNA, and second to allow the attachment of DNA polymerase and other proteins forming the transcription apparatus, which unzips, transcribes and rezips the DNA of the gene. The PIC is a multi-protein complex, which includes general transcription factors, such as TFIIA - H, and DNA polymerase II. Many genes have a TATA box motif, which provides a binding site for transcription factors, located approximately 25 base pairs upstream of the transcription start site (TSS). For genes lacking a TATA box, prior to the TSS there is an initiator region and this determines the strength of the promoter. In addition to this promoter region, genes may have other regulatory regions, which are usually (but not always) located 5’ to the promoter region and may be thousands of base pairs away from the TSS. Depending on the transcription factors that bind them, these regions may enhance or inhibit protein expression (39, 40).

Early characterisation of the PrP gene (*prnp*) found that both prion isoforms (PrP^c^ and PrP^sc^) were encoded by the same gene, and that no evidence of gene rearrangement was seen when comparing scrapie infected tissues to uninfected tissues (41). This provided further evidence that the transition from PrP^c^ to PrP^sc^ was the result of post translational modification rather than a result of gene rearrangement or alternative splicing. The original study (41) described the hamster *prnp*. The hamster gene was composed of two exons, the first was located 10kb upstream of the second, which encoded the entire uninterrupted open reading frame (ORF). In addition, several features typical of housekeeping genes were identified in the promoter (the region of DNA upstream of the putative transcription start site) including a GC rich region lacking a TATA box and three repeats of a sequence encoding Sp1 binding sites. Sp1 factors bind to these GC rich regions and, by direct protein–protein interaction of factors binding adjacent sites, loops the DNA to synergistically activate the gene by directly or indirectly recruiting proteins of the transcription apparatus (for review see 42).

The structure of the prion gene has now been characterised in several species. In contrast to the initial study describing the two exon structure of hamster *prnp*, in most species it was found that the gene includes three exons with the third, final exon encoding the entire ORF (43-45). The preceding two exons encode an mRNA 5’ untranslated region (46). The human gene, however, was shown to have the same structure as the hamster gene, with two exons and one intron present, and the second exon encoding the ORF (47).

Following the determination of the three exon structure of mRNA from other mammalian species, Li & Bolton (48) discovered the presence of a further exon in hamster mRNA, which showed significant sequence homology with exon 2 from other mammalian species. This data did not disprove the original finding that hamster mRNA was composed of two exons but instead showed there were two splice variants of the hamster mRNA, one with and one without the middle exon 2. In addition this study showed that the two variants were expressed at different levels in different parts of the brain, with the two-exon structure favoured over the three exon structure, and that expression was altered during infection causing an increase in the levels of the three exon structure. This indicated that exon 2 may be involved in the regulation of PrP expression. The equivalent exon has also been found in humans (49). This sequence has not been found in the mRNA transcript but sequence analysis of the gene, when compared to that of the mouse and sheep genes, shows a conserved region corresponding to the exon 2 sequence, which is flanked by splice sites. Since this exon has still not been identified in PrP mRNA transcripts it is thought that, like the hamster mRNA three exon transcript, expression levels of this mRNA variant may be considerably lower than its two exon counterpart and so remain undetected. An alternative theory is that exon 2 is redundant for the regulation of PrP expression, and so its loss from the mRNA transcript is of no consequence.

**Figure 1:**
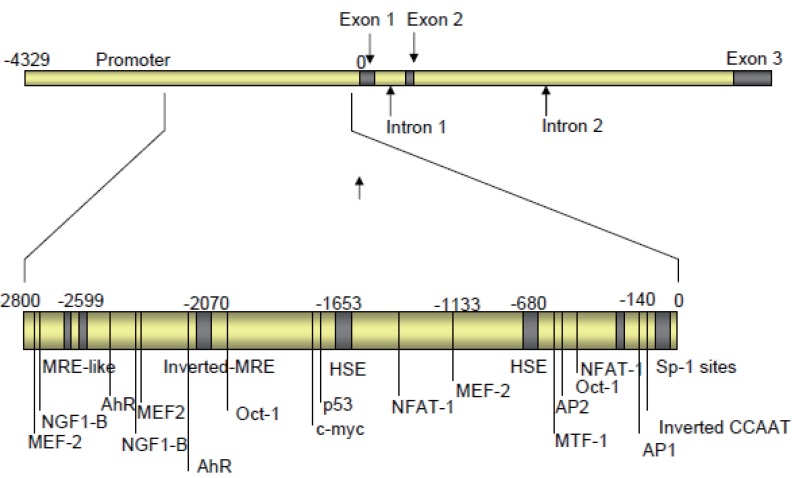
Gene structure of *prnp*. Shown are the documented regulatory domains of the promoter region. Also shown are the location of response elements within *prnp*. Exact location will vary from species to species. Some of the transcription factors indicated are: MTF-1 (metallothionein-1 DNA binding element), Oct-1 (octamer binding factor), NGF1-B (steroid hormone receptor family member), c-myc (proto-oncogene), MEF-2 (transcriptional activator), NFAT-1 (associated with Fos/Jun activation), AhR (anyl hydrocarbon receptor), MRE (metal response element) and HSE (heat shock response elements). The location of the sites shown is based upon published data on Prnp and analysis using the online Transcription Element Search System (University of Pennsylvania).

In agreement with the initial study, all characterised prion promoters lack a TATA box, are rich in GC sequences, and include Sp-1 binding sites. Other features of the prion promoter that have been identified include four highly conserved motifs, AP-1 and AP-2 binding sites, and an inverted CCAAT motif (not present in the bovine or ovine promoter), which can enhance or inhibit promoter activity dependant on what transcription factors bind to them (see Figure 1) (43, 44, 45, 46). Promoter activity is further regulated by chromatin conformation, as shown by Cabral *et al*. (50), who demonstrated the use of a histone deacetylase inhibitor could, though chromatin disassembly, cause a significant increase in promoter activity and an increased response to factors known to modulate the PrP promoter including nerve growth factor (NGF) and cAMP.

### The Role of Intron 1

In addition to the above promoter domains influencing expression, intron 1 is required for full activity of the promoter (43). This study, using the bovine gene, found that although intron 1 contributed to promoter activity, it was not an enhancer region. The authors suggest that intron 1 may be involved in tissue specific expression or synergistic control of the promoter. Intron 1 was not required for the full activity of the rat promoter. An area of promoter activity has been identified adjacent and 5’ to exon 2 of the murine gene, and the intron 1 sequence was shown to contain two areas of potential transcription factor binding sites, however an mRNA transcript lacking exon 1 was not found (51). The implication is that this region may not have its own active TSS or that mRNA levels are so much lower than the full length mRNA that only this is detected. In addition, this area was shown to contain elements capable of suppressing activity of both the promoter and the intron 1 regulatory region.

The role of intron 1 in promoter control is further supported by evidence from Premzl *et al*. (52). This study identified thirty-three potential regulatory elements within the non-coding region of human, mouse, bovine, ovine, and tamar wallaby *prnp*, seven of these elements occurred in all five species. Some were located in intron 1 and some in intron 2, indicating that the intron 2 sequence may also have regulatory function. Of the motifs conserved across all species tested were regions expected to bind myeloid zinc finger-1 (MZF-1), myocyte enhancer factor-2 (MEF2), Octamer 1 (Oct-1), myelin transcription factor 1 (MyT1), and nuclear factor of activated T-cells (NFAT).

The finding of control elements inside intron 1 is not unusual. Many genes have conserved regulatory regions located within the intron sequences, which may contribute to the control of gene expression (53-56). Control elements are preferentially found located in the 5’ introns with less control elements found in the introns located further down stream. The enhancer effects of these control regions are so essential that some genes will not be expressed in their absence. In addition, these control elements may be involved in the tissue specific expression of the gene product.

### Exogenous Factors Modulating Prion Protein Expression

Heat shock has been shown to up-regulate both *prnp* mRNA and protein expression by 1.5 to 2.5 fold in human cells, and two heat shock elements have been detected in the rat prion promoter (57). Using repressors of transcription it was shown that this up-regulation occurs at a transcriptional level and is abolished or reduced when the heat shock elements are absent or mutated (58). In addition, heat shock proteins are up-regulated in the end stages of prion disease (59), indicating that PrP mRNA levels may also be up-regulated causing an increase of protein expression. Since disease progression is directly related to PrP^c^ expression this may, in part, account for the rapid progression of the disease in the end stage.

Using hyperbaric oxygen as a model of oxidative stress, it has been shown that oxidative stress can up-regulate both PrP mRNA and protein levels in mouse neuroblastoma cells (60). In this study up-regulation of heat shock protein 70 (hsp70) was also seen, indicating that oxidative insults may up-regulate *prnp* activity by the modulation of heat shock proteins. These would activate the promoter by binding to the previously described heat shock elements. Stress caused by hypoglycaemia has also been found to up-regulate PrP mRNA and protein through a heat shock mediated response, which may involve c-Jun N-terminal kinase (JNK), a signalling kinase that modulates transcription factor production and activity (61).

Nitric oxide (NO) is a highly reactive radical used as a signalling molecule by cells. It has been found that PrP^c^ is up-regulated both at the mRNA and protein level in response to direct insult with NO or indirectly in response to lypopolysaccharide treatment, which caused up regulation of neurone specific nitric oxide synthase (nNOS) (62). Investigation into the mechanism of this up-regulation revealed that it was mediated by a signalling pathway utilising guanylyl cyclase, MEK, and p38 MAPK signalling proteins. This pathway is involved in cellular survival (63, 64), implying PrP^c^ may be up-regulated as a cell survival response.

**Figure 2:**
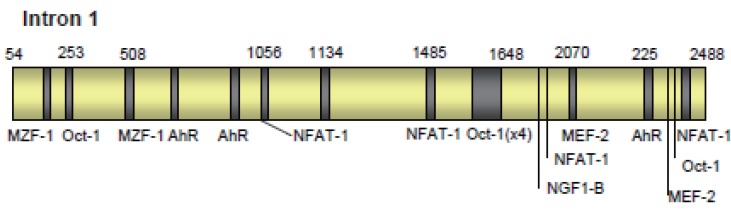
The location of response elements within Intron1. Exact location will vary from species to species. See Figure 1 for detail of sites.

Copper up-regulates PrP^c^ at the protein level (65). Copper induced up-regulation of *prnp* is thought to involve activation of the promoter by metal response elements (MREs) or MRE-like sequences. Within the bovine *prnp*, an inverted MRE at position –2070 and two MRE-like elements at positions –2653 and -2599, differing from the MRE sequence by just one nucleotide, have been located by sequence analysis (66). However deletion studies using the bovine promoter find that the removal of these elements does not fully inhibit the ability of copper to up-regulate expression and activation was not mediated by MRE-binding transcription factor-1 (MTF-1), indicating that there may be other motifs and factors involved in regulation of the gene in response to copper. This, in turn, may indicate that the ability of the gene to become activated in response to copper is so important that several regions of the gene sequence are functional for copper induced regulation. A further finding of the study is that copper induced up-regulation is not mediated by the induction of a general stress response, which would activate the promoter by the induction of heat shock proteins. This implies a copper specific response. The locations of elements modulating PrP expression are shown in Figure 2.

The study described above is in contrast to a study by Toni *et al*. (67), who find that copper down-regulates *prnp* activity at both the mRNA and protein level. The most likely reason for the difference, and a point raised by the Varela-Nallar study (66), is that response to copper is cell line specific. Since PrP^c^ expression is variable in dissimilar tissues it is most likely that genetic control mechanisms operate differently within these tissues, and both studies agree that copper is able to influence the activity of *prnp*.

All-Trans Retinoic Acid (ATRA) is a drug used for the treatment of promyelocytic leukaemia. It is an effective treatment, sending the disease into remission by inducing terminal differentiation of the malignant clone. ATRA has been found to down regulate PrP^c^ expression (68). It is possible that ATRA exerts its effect on the PrP promoter by modulation of transcription factors involved in cellular differentiation. Myt1 is a transcription factor involved in cellular differentiation and, as mentioned above, a highly conserved Myt1 binding element is found in *prnp* from several species (52).

**Figure 3:**
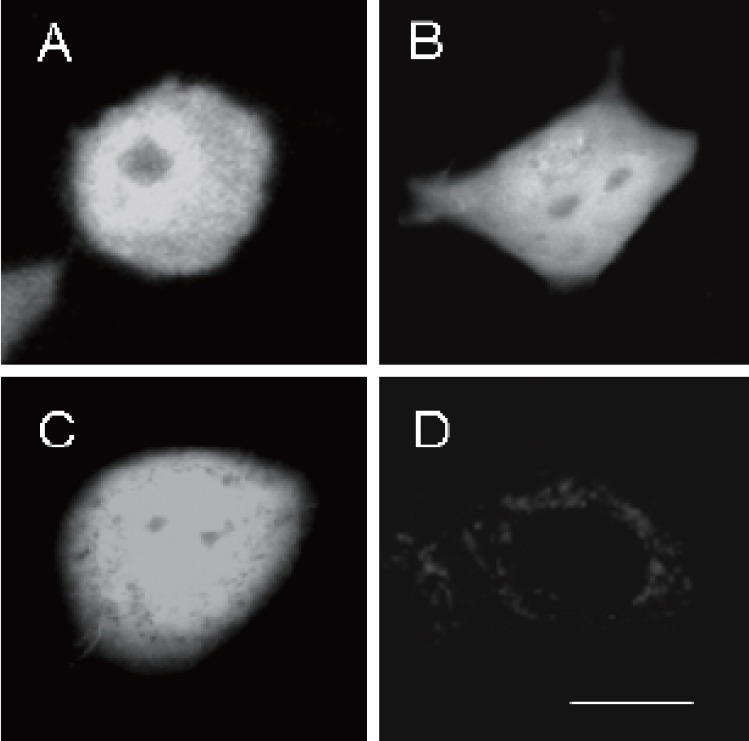
Expression of the prion protein promoter in different cell types. The neuronal F21 cell line (A), Cos cells (B), SHSY5Y cells (C) and the G8 glial cell line (D) were all transfected with a reporter construct. The construct contained the prnp promoter driving a destabilised GFP (green fluorescent protein) with a half life of 2 hours. The cells were observed under confocal microscopy. As can be seen the neuronal cell lines show high prnp promoter while the G8 cell line shows almost no prnp promoter activity. Scale bar=10 m.

An alternative mode of action of ATRA could be activation of the retinoic acid receptor (RAR) allowing it bind to Retinoic Acid Response Elements (RAREs) either as a homodimer or as a heterodimer with Retinoic X receptor (RXR). The RAR can act as an enhancer or a repressor of transcriptional activity depending on the orientation in which it binds to DNA (69). Once bound, protein interactions recruit transcription factors such as AP-1 and AP-2. However, as yet no RAREs have been identified in the *prnp*, indicating that ATRA may be more likely to exert a secondary effect through activation of transcription factors rather than direct interaction of RAR with *prnp* (Figure 3).

ATRA was also found to down regulate PrP expression by (50), who propose that down regulation of PrP results through the activity of AP-2 transcription factors, their interaction with AP-1 transcription factors and the repression of c-fos, which is also involved in modulating gene expression. Similarly ATRA induces expression of MKP-1, which inactivates members of the mitogen activated protein (MAP) kinase family, including JNK. In turn, this results in reduced activation of c-Jun and c-fos and potent inactivation of AP-1 (70). PrP may be down-regulated by ATRA using these pathways in the opposing way to that suggested for up-regulation by copper. The exogenous factors regulating PrP expression are listed in Table 1.

**Table 1:** Exogenous factors modulating PrP^c^ expression levels

Factors up-regulating PrP expression	Factors down regulating PrP expression

Copper* (66)	Copper* (67)
Heat Shock (57,58)	ATRA (50,68)
Hyperbaric Oxygen (oxidative stress) (60)	
Hypoglycaemia (61)	
Nitric Oxide (62)	
Stroke (78)	
Nerve Growth Factor (50)	
cAMP (50)	
Helicobacter pylori, Gastrin, PGE_2_, IL-1B (79)	

### Potential Therapeutic Intervention in the TSEs

Given the correlation of disease progression with PrP^c^ levels, factors that down-regulate expression may have a potential role in disease therapy or management. It might be possible that ATRA and other similarly reacting agents could be used to slow the progression of the disease.

During the early stages of disease progression endogenous PrP^c^ levels may become depleted due to conversion to PrP^Sc^. The loss of function associated with this change, as well as altered metal ion concentrations, could result in the cell up-regulating *prnp*. This may cause increased levels of PrP^c^, increased substrate for the conversion reaction, and a further increase in promoter activity causing the disease to progress in an exponential fashion. Down-regulating the promoter and breaking the cycle could be a very important method of therapeutic intervention. In this way, agents that might alter *prnp* regulatory pathways resulting in PrP^c^ protein down-regulation may have a role in disease management. Copper modulates *prnp* activity, but this reaction appears variable across different cell lines (66, 67), so it is unclear if copper modulation of *prnp* could be useful. Copper chelators would have limited application in disease management due to the importance of copper in various enzyme systems including anti-oxidant enzyme such as superoxide dismutase (SOD).

ATRA down-regulates *prnp* and PrP^c^ expression (50, 68). It is likely to exert its effect by alteration of several transcription mechanisms, including modulation of transcription factors involved in cellular differentiation and alteration of chromatin structure. Unfortunately the potential side effects to the regulation of other genes may make this drug unsuitable for use in TSE patients. Serious side effects can be seen in those treated with ATRA for promyelocytic leukaemia, including retinoic acid syndrome which may lead to organ failure and death (71). These side effects can be a result of the underlying disease pathology; nonetheless it is beneficial to continue the search for a more benign treatment for TSE patients.

Di-methyl sulphoxide (DMSO) has previously been suggested as a potential therapeutic agent for TSE treatment due to findings that the accumulation of PrP^Sc^ and disease progression was delayed in DMSO treated hamsters (72). DMSO is a versatile molecule. It can act as an oxygen radical scavenger and so has been implicated in protection from oxidative stress. However, the incorporation of oxygen radicals into the DMSO molecule initiates its breakdown, during which process further radicals are released. These are mostly methyl radicals (although at higher concentrations methyl sulphoxide radicals are produced by pyrolysis) and in an aqueous system can react with oxygen to form peroxyl radicals, thus inducing oxidative stress (73, 74). In addition to these qualities, DMSO can also initiate apoptosis by collapsing the mitochondrial membrane potential and the subsequent activation of caspases 3 and 9. It can induce cellular differentiation of some malignant clones, and alter expression of various cell surface antigens (75). The oxidative stress produced by DMSO or its other effects may up-regulate PrP^c^. An agent that up-regulates PrP^c^ expression would be expected to provide more substrate for conversion and so is likely to be unsuitable for use in disease therapy. This highlights the difficulty in using a multifaceted agent such as DMSO. Agents with a more unambiguous mode of activity may be more suitable for trial.

Factors that could maintain low cellular levels of oxidative stress may down-regulate the activity of *prnp* and result in a decrease in the quantity of PrP^c^ protein produced by the cell. Antioxidant replacement, in addition to potentially down regulating PrP^c^ protein expression, could also counteract decreased activity of cellular SODs seen during disease pathogenesis due to aberrant metal metabolism. Mitochondrial SOD/SOD2 synthetic mimetics that can cross the blood brain barrier have been shown to protect SOD2 null mice against spongiform encephalopathy that results due to the null phenotype and may have a potential use in treatment of TSEs (76). Agents such as this should be further investigated for potential therapeutic uses.

## CONCLUSION

The control of PrP^c^ expression is complex. It involves not only the promoter and regulatory regions preceding the promoter, but also intron 1. These control regions may provide targets for controlling PrP^c^ expression levels. Since PrP^c^ is required for disease establishment, and the rate of disease progression is directly related to the level of endogenous PrP^c^, factors able to down regulate PrP^c^ expression levels could have a role in the management of the TSEs. Such a strategy would require the discovery of compounds that could not only effect the expression of PrP but also that could be delivered into the CNS and enter the cells where PrP conversion occurs. Drug delivery could be the main barrier to develop a strategy related to regulating PrP expression to combat prion diseases. However, such concerns are barriers for most dug based therapeutic strategies. Therefore, a new approach, such as gene regulation, could increase the possibility of finding the appropriate target for a via therapeutic strategy for TSEs.
